# Examining the Hierarchical Influences of the Big-Five Dimensions and Anxiety Sensitivity on Anxiety Symptoms in Children

**DOI:** 10.3389/fpsyg.2019.01185

**Published:** 2019-06-04

**Authors:** Erika Wauthia, Laurent Lefebvre, Kathy Huet, Wivine Blekic, Khira El Bouragui, Mandy Rossignol

**Affiliations:** ^1^Department of Cognitive Psychology and Neuropsychology, Faculty of Psychology and Education, University of Mons, Mons, Belgium; ^2^National Fund for Human Research (FRESH), National Fund for Scientific Research, Brussels, Belgium; ^3^Interdisciplinary Research Center in Psychophysiology and Cognitive Electrophysiology, Mons, Belgium; ^4^Laboratory of Phonetics, Research Institute for Language Sciences and Technology, Faculty of Psychology and Education, University of Mons, Mons, Belgium; ^5^Laboratory C2S, University of Reims Champagne-Ardenne, Reims, France

**Keywords:** Big-Five personality, neuroticism, anxiety sensitivity, anxiety symptoms, children, vulnerability factors

## Abstract

Anxiety sensitivity (AS), namely the fear of anxiety symptoms, has been described as a precursor of sub-threshold anxiety levels. Sexton et al. (2003) posited that increased AS would arise from an elevated neuroticism and that both would act as vulnerability factors for panic disorder (PD), obsessive-compulsive disorder (OCD), and generalized anxiety disorder (GAD) symptoms. Accordingly, this study aimed to (1) evaluate the applicability of this model to a pediatric population and (2) examine the influences of the other Big-Five personality dimensions on the four lower-order dimensions of AS (cognitive, physical, control, and physical) and on social phobia (SP), separation anxiety disorder (SAD) and depression symptoms. 200 children (104 girls) aged between 8 and 12 years old (mean age = 132.52 months, *SD* = 14.5) completed *the Childhood Anxiety Sensitivity Index* (Silverman et al., 1991), *the Big Five Questionnaire for Children* (Barbaranelli et al., 2003), and *the Revised’s Children Anxiety and Depression Scale* (Chorpita et al., 2000). Regression analyses confirmed that AS and neuroticism together significantly predicted the presence of PD, OCD, and GAD symptoms but also SP, SAD, and depression symptoms. Moreover, neuroticism interacted with extraversion, conscientiousness and agreeableness to significantly predict SP, GAD, and depression. Surprisingly, the global AS score was only predicted by agreeableness, while AS dimensions also specifically related to openness. Finally, AS dimensions did not predict the presence of specific anxiety symptoms. To conclude, the predicting model of anxiety symptoms in children sets neuroticism and AS on the same level, with an unexpected influence of agreeableness on AS, raising the importance of other trait-like factors in the definition of such models. Moreover, AS should be considered as a unitary construct when predicting the presence of anxiety symptoms in children. Future interventions must consider these associations to help children detect and recognize the symptoms of their anxiety and help them to interpret them correctly.

## Introduction

While a large number of youths report excessive anxiety-related emotional and behavioral responses (e.g., avoidance) and anxiety-related cognitive patterns (American Psychiatric Association [APA], 2013), only up to 20% of them meet the criteria for an anxiety disorder diagnosis (Costello et al., 2004). Moreover, subthreshold anxiety levels interfere with children’s general well-being, developmental social skills and social life (Pine et al., 1998; Kendall et al., 2001; Kendall and Treadwell, 2007; Mazzone et al., 2007; Kessler et al., 2012). Notably, they are associated with impaired memory and cognitive performances (Bulbena and Berrios, 1993; Daleiden, 1998) and consequently have precarious impacts on the academic adjustment of children (Fergusson and Woodward, 2002; Wittchen and Fehm, 2003; Mazzone et al., 2007; Beesdo et al., 2009; Thapar et al., 2012). Subthreshold anxiety levels also have a high comorbidity with other mental disorders, such as depressive moods, and are associated with an increased prevalence of mental disorders in adulthood (Fergusson et al., 2005; Bittner et al., 2007; Copeland et al., 2009; Wolitzky-Taylor et al., 2014) and with an enhanced suicide risk (Balázs et al., 2017). Indeed, the presence of sub-threshold panic disorder (PD), obsessive-compulsive disorder (OCD), and social phobia (SP) predict the full onset of these disorders later on (Wolitzky-Taylor et al., 2014). Therefore, a major concern is identifying children that are at high risk of developing anxiety symptoms and preventing deleterious consequences. This can be done by gaining a better understanding of the etiological factors of sub-threshold anxiety levels.

Recently, increasing attention has been given to anxiety sensitivity (AS) that constitutes a stable, trait-like cognitive vulnerability factor for anxiety disorders (Olatunji and Wolitzky-Taylor, 2009). AS – also known as the fear of anxiety – refers to the tendency to interpret anxiety symptoms (including somatic sensations) as signals of social, psychological or physical catastrophe susceptible to cause illness, embarrassment or additional anxiety (Reiss et al., 1986). For example, an individual with elevated AS levels might be more likely to interpret a racing heart as a precursor to a heart attack or will fear to sweat in public because of his concerns about negative social evaluations. If AS is a dispositional variable, it describes a specific tendency to fearfully respond to one’s own anxiety symptoms (Olatunji and Wolitzky-Taylor, 2009); which is thus distinguishable from trait anxiety which refers to a tendency to respond with fear to a wide range of stressors (Spielberger, 1989). In other words, unlike individuals with high levels of AS, individuals with high trait anxiety will not respond to anxious symptoms unless they identify them as a threat (McNally, 1989).

Anxiety sensitivity has been conceptualized as an higher-order factor hierarchically organized by several lower-order factors (Cox et al., 1996; Zinbarg et al., 1997; Taylor and Cox, 1998; Muris, 2002). In children and adolescents, AS is measured with the Childhood Anxiety Sensitivity Index (CASI, Silverman et al., 1991) that is an age-downward modification of the Anxiety Sensitivity Index (ASI; Peterson and Reiss, 1992; Zinbarg et al., 1999; Taylor et al., 2007) initially developed for adults. This inventory investigates three distinct dimensions of AS, namely Physical Concerns (fear of physical symptoms of anxiety; i.e., “it scares me when my heart beats rapidly”); Cognitive Concerns (fear of loss of cognitive control; i.e., “when I’m nervous, I worry that I may be mentally ill”) and Social Concerns (fear of publicly observable anxiety symptoms; i.e., “it embarrasses me when my stomach growls”). Recently, Stassart and Etienne (2014) proposed to add a fourth lower-order factor relating to Losing Control Concerns (i.e., “Other kids can usually tell when I feel shaky”).

The CASI has proven to be a reliable and valid questionnaire for measuring AS in both clinical and non-clinical pediatric populations (Silverman et al., 1991; Rabian et al., 1999). Using this instrument, studies revealed that AS is typically higher in children meeting diagnosis criteria of anxiety disorders and in highly anxious children as compared to healthy control children (Rabian et al., 1993; Vasey et al., 1995; Joiner et al., 2002; McLaughlin et al., 2007). However, AS scores do not distinguish between children reporting anxiety disorders and children with externalizing disorders, probably due to comorbid diagnoses (Rabian et al., 1999).

As already mentioned, it has been shown that AS determines the development and severity of a large variety of fears and anxiety disorders (Taylor et al., 1992) and seems particularly prominent in those based on somatic signals, as social anxiety or PDs. Notably, Taylor et al. (1992) compared the levels of AS across DSM-III anxiety disorders and revealed that AS was elevated in all diagnostic groups, in comparison with normal controls, except for simple phobia. Similarly, Kearney et al. (1997) found higher levels of AS in children and adolescents with clinical levels of PD. Eley et al. (2004) reported similar results in adults, which showed an enhanced ability to perceive internal physiological cues and a tendency to fear them. Higher levels of AS were also observed in youths with SP and associated with the fact that they interpret their enhanced physiological arousal as a visible cue of anxiety and a potential source of embarrassment in social situations (Eley et al., 2004; Anderson and Hope, 2009).

Deacon and Abramowitz (2006) examined the relationships between the three dimensions of AS and the type of anxiety disorder in adults. They reported higher AS scores in anxious patients than in healthy undergraduate students and higher scores in patients with PD than in patients with SP and generalized anxiety disorder (GAD). Their analyses confirmed that the physical concerns’ dimension was related to PD supporting previous results (Zinbarg et al., 1997, 2001; Hayward et al., 2000; Blais et al., 2001) and the idea that fear of physical sensations can contribute to the panic-related psychopathology. Secondly, they outlined a near-exclusive relationship between the social concerns’ dimension and SP. Finally, the cognitive concerns dimension can account for elevated AS in patients with OCD (Cox et al., 1999; Sexton et al., 2003) although conflicting results have been highlighted (Deacon and Abramowitz, 2006).

Prospective studies indicated that AS constitutes a predictor of the future occurrence of anxiety symptoms and anxiety disorders (Schmidt et al., 1997, 1999; Hayward et al., 2000; Schmidt et al., 2006). Conducting longitudinal studies with a follow-up assessment 1 year after the baseline, Weems et al. (2007) and Schmidt et al. (2010) found that AS predicted the development of various symptoms of anxiety disorder in early adolescence and could be considered as a cognitive risk factor. The predictive role of AS was also reported among children and adolescents for PD (Lau et al., 1996; Reiss et al., 2001), OCD (Calamari et al., 2001), and PTSD symptoms (Kılıç et al., 2008).

Cognitive models of anxiety disorders offer a theoretical framework for the relationship between AS and the subsequent development of anxiety. Notably, Wells and Leahy (1998) proposed that, if anxiety symptoms are initially caused by biological factors, their persistence are the consequence of a misinterpretation of internal/external events. Indeed, events such as bodily sensations caused by a postural change in blood pressure, tiredness, excitement or stress are taken as a sign of an immediate catastrophe such as dying, suffocating, having a heart attack/seizure, fainting, collapsing, losing one’s mind or losing control (Wells, 2013). Once misinterpretations developed, a shift in selective attention occurs and anxious individuals become self-focused on negative feelings and symptoms. In order to advert those, engage themselves in safety/coping behaviors that will maintain and exacerbate symptoms through four mechanisms (Wells and Leahy, 1998). First, anxiety persists because the non-occurrence of the catastrophe is attributed to the used behavior. Second, the use of certain safety behaviors will intensify or prolong unwanted symptoms (e.g., focusing on heart rate will consequently increase it). Third, some safety behaviors increase self-focused attention -as the person focuses attention inward to monitor and gauge the effectiveness of those behaviors- that amplifies awareness of their symptoms. Fourth, safety behaviors can interfere with the adaptation of the anxious individuals in everyday situations. According to this model, AS would amplify preexisting anxiety levels to the extent that individuals with high levels of AS may misinterpret physical sensations as danger signals and as a result experience elevated levels of anxiety (Olatunji and Wolitzky-Taylor, 2009).

Anxiety sensitivity’s levels can be modulated by the dimensions of the Five-Factor Model of Personality (Big Five) (McRae and Costa, 1996) and notably by neuroticism. Also known as emotional instability or negative affectivity (Watson and Clark, 1984), neuroticism refers to the tendency to experience negative emotions and the poor ability to cope with stress. Despite some conceptual differences, reviews suggested that neuroticism and trait anxiety are highly similar constructs (Zinbarg and Barlow, 1996; Barlow, 2000; Sexton et al., 2003); they arise from genetic influences and from early childhood learning and they comprise cognitive and psychological features or tendencies to readily perceive threat, and to be readily aroused (Craske, 1999; Sexton et al., 2003).

Studies revealed that elevated levels of AS were positively correlated to an increased neuroticism (Cox et al., 1999). This is consistent with previous studies using the Big Three model of personality (Tellegen, 1985) showing that AS was positively correlated to negative emotionality (NE) (Arrindell, 1993; Lilienfeld, 1997). AS appears to be negatively associated to extraversion, which refers to one’s quantity and intensity of interpersonal interactions and positive emotions, and with conscientiousness that is characterized by a disciplined striving after goals and a structured adherence to principles (Costa and McCrae, 1990; Borger et al., 1996; Brandes and Bienvenu, 2006). Nowadays, while neuroticism and extraversion are the only significant predictors of AS (Borger et al., 1996), studies investigating its relationships with other personality dimensions such as openness – that includes intellectual curiosity, the need for variety and non-dogmatic attitudes–and agreeableness that involves trust, altruism and sympathy, are scarce (Bienvenu and Stein, 2003; Bienvenu et al., 2004), especially in children.

Literature highlighted that these personality dimensions also appear to be associated with anxiety disorders, as demonstrated in adults (Clark et al., 1994; Brown, 2007; Kotov et al., 2010; Naragon-Gainey et al., 2014). Notably, Bienvenu et al. (2001) evidenced higher scores of neuroticism in adults who reported a history of SP, agoraphobia or PD. Individuals with a history of SP and agoraphobia also presented lower scores of extraversion, supporting previous results (Solyom et al., 1986; Watson et al., 1988; Trull and Sher, 1994; Brown et al., 1998; Naragon-Gainey et al., 2014; Newby et al., 2017). Higher scores of neuroticism have been found in patients with OCDs and post-traumatic stress disorders (PTSD) (Trull and Sher, 1994; Samuels et al., 2000), but PTSD patients also showed lower scores of extraversion, agreeableness and conscientiousness (Trull and Sher, 1994). In children, longitudinal studies also revealed that high levels of neuroticism and lower levels of extraversion were predictors of higher levels of anxiety (Ehrler et al., 1999; Muris et al., 2004; Vreeke and Muris, 2012). Interestingly, Naragon-Gainey et al. (2014) suggested that the development of social anxiety disorder may result from the interaction between low levels of extraversion and increased AS level.

Therefore, neuroticism seems related to both constructs of AS and anxiety disorders. Brown et al. (1998) defined a model in which neuroticism would be a higher-order factor that has a direct causal influence on various types of anxiety disorders. According to this model, neuroticism would operate with another factor, positive affectivity (PA) which contributes to the development of depressive affects. Taylor (1998) refined this model by postulating the existence of a hierarchical model in which fears are the product of various etiological factors with different levels of specificity. Some factors are disorder-common factors (i.e., neuroticism) and others are disorder-specific factors such as AS. Extending this model, Sexton et al. (2003) combined the influence of AS and neuroticism on the development of anxiety disorders. In their model, neuroticism acts as a general vulnerability factor that directly prompts the onset of anxious symptoms, namely worry, panic symptoms, health anxiety and OCD symptoms. In parallel, neuroticism mediates the development of anxiety sensibility which in turn intensifies the four categories of anxious symptoms. To sum up, neuroticism may constitute a general vulnerability while AS would be a specific etiological variable in the occurrence of specific anxiety complaints. Norton et al. (2005) replicated this hierarchical model in patients suffering from clinical levels of anxiety disorders and obtained highly consistent results.

Accordingly, the first aim of this study was to test the applicability of Sexton et al.’s (2003) hierarchical influences of neuroticism and AS on anxiety symptoms in children between 8 and 12 years of age. We hypothesized that high levels of neuroticism would directly predict increased AS levels and increased PD, OCD, and GAD symptoms and that high AS levels would also be directly associated to these symptoms.

The second aim of this study is the examination of a second, more elaborated, model derived from the first one. As already done by Norton et al. (2005) and Norton and Price (2007), we tested paths for SP, SAD, and depressive symptoms which are highly prominent symptoms in children (Costello et al., 2005; Beesdo et al., 2009). Furthermore, we added the influence of other Big-Five personality traits since some associations had already been shown with extraversion in adults (Bienvenu and Stein, 2003; Bienvenu et al., 2004) while other personality dimensions such as conscientiousness, openness and agreeableness had not been investigated. We expected that extraversion as the positive corollary of neuroticism would have direct and indirect significant effects on all anxiety symptoms and on AS levels. Due to the fact that OCD may be conceptualized as a maladaptive version of conscientiousness and that some conscientiousness-related traits converged with OCD in adults (Samuels et al., 2000), we also postulated that conscientiousness would have direct effects on OCD symptoms in school-aged children.

The third objective of this study was to examine this extended hierarchical model, decomposing AS into its four lower-order factors and to investigate associations between these factors, personality dimensions and anxiety symptoms. Since PD is defined by the occurrence of panic attacks whose physical manifestations are significant (e.g., palpitations, sweating, and shaking) (Craske et al., 2010), we hypothesized that PD symptoms would be related to higher scores on the physical concerns dimension. Given that SP is defined as the excessive and persistent fear of (one or more) social or performance situations involving exposure to others (Furmark, 2002; Schneier, 2006), we expect that SP symptoms would be associated to higher scores on the social concerns dimension. The prominent presence of worries in GAD (Chorpita et al., 1997; Weems et al., 2000) allowed us to posit that GAD symptoms would be associated with cognitive concerns and (4) the rigidity of thoughts characterizing OCD symptoms led us to hypothesize that OCD symptoms would be associated with cognitive and losing control concerns.

## Materials and Methods

### Participants

Ten primary schools in the Mons region (Belgium) were contacted and agreed to participate in this study. Inventories were distributed to parents who had to sign an agreement before their child’s participation. 250 parents responded positively and 200 Caucasian children (104 girls) aged from 8 to 12 (mean age = 132.55 months; *SD* = 14.5) completed all the inventories. All children were free from learning disorders, neurologic or psychiatric conditions as assessed with the Child Behavior Checklist (Achenbach and Edelbrock, 1983; Fombonne et al., 1988). The study was carried out in accordance with the recommendations of the Ethic Board of the Faculty of Psychology and Education of the University of Mons. The protocol was approved by the committee. All participants and their legal guardian gave written informed consent in accordance with the Declaration of Helsinki.

### Measures

*Childhood Anxiety Sensitivity Index* (Silverman et al., 1991; Stassart et al., 2013) - The CASI is a self-report inventory including 18 items scored on a three-point Likert scale ranging from 1 “not at all” to 3 “very much.” On each item, children have to rate the extent to which the experience of anxiety will have negative consequences. Example items are “It scares me when my heart beats fast,” “It scares me when I feel nervous,” and “It scares me when I feel shaky.” Test-retest reliability has been reported at 0.79 in clinical and at 0.76 for non-clinical samples (Bilgiç et al., 2013). The French version of CASI has a satisfactory validity and good internal consistency, with a Cronbach’s alpha of 0.87 (Vanasse et al., 2010; Stassart and Etienne, 2014). The CASI allows the calculation of four lower-order dimensions: cognitive, social, physical and fear of losing control (Stassart and Etienne, 2014). Cronbach’s alphas of the separate dimensions of the AS ranged from 0.33 to 0.82 (Stassart and Etienne, 2014).

*Big Five Questionnaire for Children* (Barbaranelli et al., 2003; Rossier et al., 2007 *for the French version*) – The Big Five Questionnaire for Children (BFQC) is a self-reported measure consisting of 65-items used for assessing the basic personality dimensions of extraversion, agreeableness, conscientiousness, emotional instability or neuroticism and openness in youths. Each of the five dimensions is evaluated by 13 items. These items are rated on a five-point Likert scale ranging from 1 “Almost never true” to 5 “Almost always true.”

*Revised Children Anxiety and Depression Scale* (Chorpita et al., 2000; Bouvard et al., 2015 *for the French version*) – The Revised Children Anxiety and Depression Scale (RCADS) is a 47-items self-reported questionnaire used to assess DSM-IV anxiety and depression symptoms in children and adolescents from 7 to 18 years old. Children have to rate how often each item applies to them; each item is scored on a three-point Likert scale from 0 “never” to 3 “always.” Separated scores are obtained for SAD, SP, GAD, PD, OCD, and depression.

### Data Analyses

#### Data Screening and Outlier Analysis

All measures were initially assessed for multivariate outliers and univariates outliers. First, the data were assessed for multivariate outliers by entering all measures into a multiple regression analysis and computing Mahalanobis distance, as suggested in the paper of Sexton et al. (2003). A chi-square cut-off of *p* < 0.001 was used as the criteria for multivariate outliers (Tabachnick and Fidell, 1996). Four multivariate outliers were identified and removed from the data set, yielding a working *n* of 196. Univariate outliers were then identified by taking the inter-quartile range 1.5 times and declaring all data points as outliers that are either this distance above the upper quartile or this distance below the lower quartile (Hoaglin and Iglewicz, 1987). Sixty-one data points were identified as univariate outliers that were consequently Windsorized, replacing the outlying data with non-outlying values (Hoaglin and Iglewicz, 1987). We conducted the Harman’s one-factor test (Harman, 1970; Podsakoff et al., 2003) to detect the presence of common method variance. The result of the unrotated factor analysis indicated one single factor that accounted for 13.87% of the variance, that allowed to conclude that the common method only have a limited effect on the relationships between measures of different constructs.

#### Sample Characteristics

A multivariate analysis of variance (MANOVA) was computed to explore potential differences between sex groups on the dependent variables used in this study. The MANOVA revealed a significant multivariate sex difference for the openness dimension of the BFQ-C [*F*(1,127) = 5.79; *p* = 0.018]. Means obtained by our sample to the five dimensions of the BFQ-C were compared to those obtained by Olivier and Herve (2015) in their validation study. Results showed that our data were similar to those obtained by a group of French-speaking children of the same age group. To assess hierarchical relationships between variables, we conducted regression-based path analyses using multiple backward regression analyses on full-saturated models. All statistical analyses were computed with SPSS 21. The alpha level of significance was set at 0.05 throughout analyses.

## Results

### Descriptive Statistics

Mean, standard deviations of the main variables are shown in Supplementary Table 1.

### Path Analyses

**FIGURE 1:**
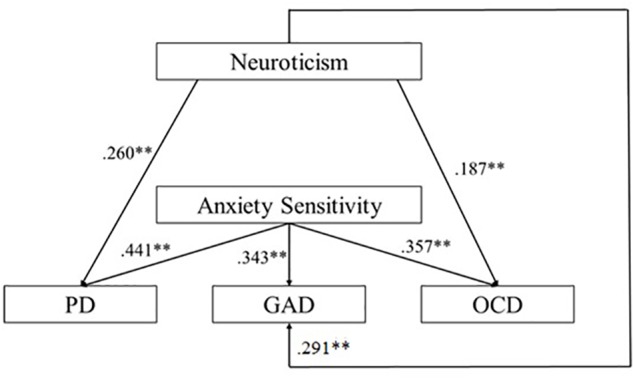
Summary of the first hypothesized model with standardized path coefficients (PD, panic disorder; GAD, generalized anxiety disorder; OCD, obsessive-compulsive disorder) (^∗^ for *p*-values < 0.05; ^∗∗^ for *p*-values < 0.01).

The first hypothesized hierarchical model was analyzed using regression-based path analyses (Kline, 1998; Sexton et al., 2003). Figure 1 presents this hypothesized model with standardized path coefficients. An examination of the effects indicated that neuroticism did not have a significant direct effect on AS [*F*(1,169) = 3.77; *p* = 0.054; *R*^2^ = 0.022]. Then, we examined effects of the vulnerability variables on the presence of PD, OCD and GAD symptoms. Analyses have shown that high levels of neuroticism and high levels of AS had significant direct effects on the presence of PD symptoms [*F*(2,169) = 25.20; *p* =< 0.001; *R*^2^ = 0.297; β = 0.260]. Results also showed that neuroticism and AS had significant direct effects on OCD symptoms [*F*(2,169) = 18.58; *p* =< 0.001; *R*^2^ = 0.182; β = 0.187]. Finally, we also found that neuroticism (β = 0.491) and AS (β = 0.343) had a significant effect on GAD symptoms [*F*(2,169) = 25.30; *p* =< 0.001; *R*^2^ = 0.233]. First hypothesized model is summarized in Supplementary Table 2.

**FIGURE 2:**
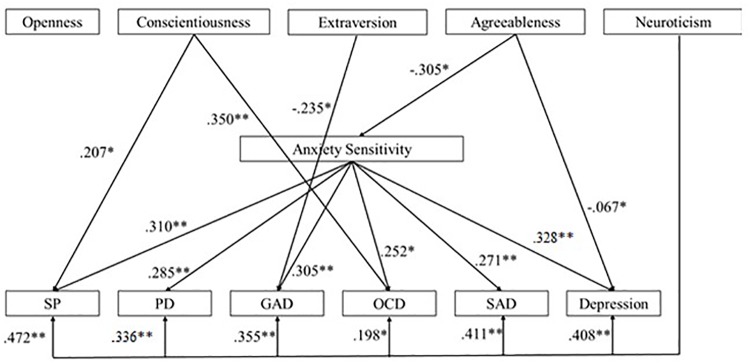
Summary of the second hypothesized model with standardized path coefficients (SP, social phobia; PD, panic disorder; GAD, generalized anxiety disorder; OCD, obsessive-compulsive disorder; SAD, separation anxiety disorder) (^∗^ for *p*-values < 0.05; ^∗∗^ for *p*-values < 0.01).

We also used backward regression-based path analyses to test the second hypothesized hierarchical model. Figure 2 presents this model with standardized path coefficients. Examination of the effects of the five personality dimensions on AS levels revealed that only agreeableness had a significant effect on AS [*F*(5,128) = 3.51; *p* =< 0.001. *R*^2^ = 0.125; β = -0.305]. Then, we evaluated the effects of personality dimensions and AS on anxiety symptoms. First, we found that high levels of conscientiousness (β = 0.207), neuroticism (β = 0.472) and AS (β = 0.310) had significant direct effects on the presence of SP symptoms [*F*(6, 128) = 11,64; *p* =< 0.001; *R*^2^ = 0.364]. Second, results showed that high levels of neuroticism (β = 0.285) and AS (β = 0.336) had direct significant effect on the presence of PD symptoms [*F*(6,128) = 6.38; *p* =< 0.001; *R*^2^ = 0.239]. Third, analyses revealed that low levels of extraversion (β = -0.235) and high levels of neuroticism (β = 0.355) and AS (β = 0.305) had significant direct effects on GAD symptoms [*F*(6,128) = 7.80; *p* =< 0.001; *R*^2^ = 0.277]. Fourth, we found that low levels of agreeableness and high levels of conscientiousness (β = 0.350), neuroticism (β = 0.198), and AS (β = 0.252) had significant direct effects on OCD symptoms [*F*(6,128) = 5.78; *p* =< 0.001; *R*^2^ = 0.221]. Analyses revealed a significant direct effect of high levels of neuroticism (β = 0.411) and AS (β = 0.271) on the presence of SAD symptoms [*F*(6,128) = 9.07; *p* =< 0.001; *R*^2^ = 0.309]. Finally, it appears that high levels of neuroticism (β = 0.408), AS (β = 0.328) and low levels of agreeableness (β = -0.067) predict depression symptoms [*F*(6, 195) = 17.09; *p* =< 0.001; *R*^2^ = 0.336]. Second hypothesized model is summarized in Supplementary Table 3.

**FIGURE 3:**
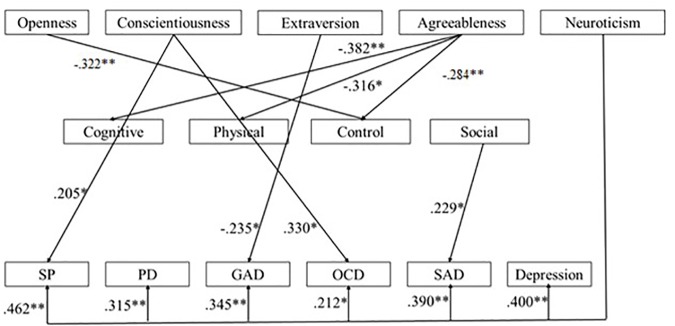
Summary of the third hypothesized model with standardized path coefficients (SP, social phobia; PD, panic disorder; GAD, generalized anxiety disorder; OCD, obsessive-compulsive disorder; SAD, separation anxiety disorder) (^∗^ for *p*-values < 0.05; ^∗∗^ for *p*-values < 0.01).

We used backward regression-based path analyses to test the third hypothesized hierarchical model. Figure 3 presents this model with standardized path coefficients. Examination of the effects of the five personality dimensions on the four AS dimensions revealed that low levels of agreeableness had significant direct effects on the cognitive [*F*(5,123) = 4.24; *p* = 0.001; *R*^2^ = 0.147; β = -0.382], physical [*F*(5,128) = 3.47; *p* = 0.006; *R*^2^ = 0.124; β = -0.316] and control [*F*(5, 128) = 3.58; *p* = 0.005; *R*^2^ = 0.127; β = -0.284].Results showed conscientiousness (β = 0.245) and openness (β = -322) also had significant direct effects on the losing control dimension [*F*(5, 128) = 3.58; *p* = 0.005; *R*^2^ = 0.127].

Finally, in this model, we investigated the effects of the five dimensions of the personality and of the four dimensions of AS on specific anxiety symptoms. Analyses revealed that high levels of conscientiousness (β = 0.205) and neuroticism (β = 0.462) had significant direct significant effects on SP symptoms [*F*(9,128) = 7.69; *p* =< 0.001; *R*^2^ = 0.368]. Neuroticism had a direct effect on PD symptoms [*F*(9,128) = 7.69; *p* =< 0.001; *R*^2^ = 0.368; β = 0.315]. Neuroticism (β = 0.345) and extraversion (β = -0.235) had effect on GAD symptoms [*F*(9,128) = 5.81; *p* =< 0.001; *R*^2^ = 0.305]. Analyses revealed that high levels of conscientiousness (β = 0.330) and neuroticism had significant effects on OCD symptoms [*F*(9,128) = 4.20; *p* =< 0.001; *R*^2^ = 0.241]. We observed that high levels of neuroticism (β = 0.390) and high levels of social concerns (β = 0.229) had a significant effect on SAD symptoms [*F*(9,128) = 6.97; *p* =< 0.001; *R*^2^ = 0.345]. Finally, we observed that depressive symptoms were predicted by high levels of neuroticism [*F*(9,178) = 10.75; *p* = 0.000; β = 0.400]. Third hypothesized model is summarized in Supplementary Table 4.

## Discussion

Previous studies conducted in adults (Brown et al., 1998; Taylor, 1998; Barlow, 2000; Sexton et al., 2003) suggested that anxiety disorders and anxiety symptoms are supposed to arise from hierarchical influences of trait-like factors as personality traits and AS. Consequently, this study intends to explore the isolate and cumulate influences of those general vulnerability factors in the development of anxiety symptoms in children between 8 and 12 years of age.

To meet this purpose, we first investigated the applicability of the Sexton et al.’s (2003) model in pediatric populations. This model suggests that neuroticism is a global vulnerability factor that directly influences the development of specific anxiety symptoms and influences the development of AS. While this model relies on a strong empirical background in adult populations (Arrindell, 1993; Borger et al., 1996; Cox et al., 1996; Lilienfeld, 1997; Taylor and Cox, 1998; Sexton et al., 2003), we failed to demonstrate a significant influence of neuroticism on AS levels in this study. With regard to this result, we hypothesize that, in children, AS may also be considered as a lower-order vulnerability factor in the same way as neuroticism. Consequently, in children, these factors should be equally treated. Another explanation would be that in our sample neuroticism will be better represented by its lower-order factor, trait anxiety. Our data suggest a strong correlation between these two variables and numerous evidences in the adult literature suggest that neuroticism and trait anxiety are highly similar constructs (Zinbarg and Barlow, 1996; Craske, 1999; Barlow, 2000; Sexton et al., 2003). Therefore, we may hypothesize that the associations between AS and trait anxiety is already strong during childhood and may broaden to the larger trait of neuroticism with age. Consequently, it would be interesting to replicate this study by adding the impact of trait anxiety in the different predicting models. Finally, it is also important to note that the absence of association between AS and neuroticism may also be attributable to our limited sample size and/or to the removal of a significant number of outlying values, underlining the need of further studies on a larger and more representative population.

Our analyses revealed that increased neuroticism scores are significant predictors of PD, GAD and OCD symptoms; confirming previous results obtained in adults by Bienvenu and Stein (2003) and Sexton et al. (2003). As predicted, AS levels also appeared to be a predictor of those anxiety symptoms in youths, confirming previous research in this domain (Lau et al., 1996; Calamari et al., 2001; Reiss et al., 2001). Relationships between AS and PD is not surprising given the vast amount of literature enhancing such effects (Kearney et al., 1997; Eley et al., 2009). Associations between AS and OCD are also understandable since, as proposed by Cox et al. (1999), AS may be represented in OCD as fear of loss of cognitive control symptoms that may stem from the experience of unwanted, intrusive thoughts, coupled with an inflated sense of responsibility for those thoughts. Accordingly, those intrusive thoughts and their negative consequences may be progressively feared. Finally, while AS had no influence on GAD symptoms in Sexton et al. (2003) study, we found here a significant effect. However, the model of Sexton et al. (2003) proposed Intolerance of Uncertainty (IU) as the vulnerability factor of excessive worry and GAD in adults, as previously shown by Koerner and Dugas (2008). Intolerance of uncertainty (IU) refers to the tendency to react negatively on an emotional, cognitive and behavioral level to uncertain situations and events (Dugas et al., 2004). While studies conducted on IU in children are still scarce (Comer et al., 2009), we may hypothesize that the significant effects of IU on GAD symptoms would appear at a later age, with the increase of mentalization abilities of children, and that the significant effect of AS would in turn decrease. However, as we choose here to focus on paths between personality, AS and symptoms, further studies must address this question adding the child form of the Intolerance of Uncertainty Scale (Comer et al., 2009).

The second aim of this study was to examine the applicability of an extended model that comprised all five personality dimensions of the Big Five model of personality (McCrae and Costa, 1999) and other symptoms frequently encountered in the young population such as SP, SAD and depression. Our results showed that AS is significantly predicted by low levels of agreeableness. Previous results in adults (Brown et al., 1998; Norton et al., 2005) showed that positive affectivity (PA), which is a common-disorder factor of neuroticism, was associated with the level of AS. However, while PA had frequently been associated to extraversion (Watson et al., 1988; Watson, 2002; McNiel and Fleeson, 2006), we found here that the agreeableness score was the significant predictor of the total AS level in children. Consequently, in our sample it appears that PA relates more to this personality dimension and was also enhanced in previous research (DeNeve and Cooper, 1998). Recent evidence suggests that low levels of agreeableness are associated with low emotional and behavioral regulation abilities due to poorer child cooperation, persistence, self-control and expressed affects (Ahadi and Rothbart, 1994; Caspi and Silva, 1995; Caspi, 1998; Laursen et al., 2002). Accordingly, diminished emotional regulation abilities could lead to a greater focus on anxiety symptoms. It would thus be possible that children with low levels of agreeableness are less able to regulate their emotions, which lead them to be more focused on, and sensitive to their symptoms. However, further studies need to replicate this result.

In this second model, analyses confirmed that higher levels of neuroticism and of AS are associated with increased anxiety symptoms, as we hypothesized with regards to previous literature (Rabian et al., 1993; Joiner et al., 2002; McLaughlin et al., 2007). However, they also showed that other frequently encountered anxiety symptoms (SP, SAD and depression) in children are predicted by increased neuroticism and AS. Those results are in line with studies conducted in adults (Bienvenu et al., 2001; Eley et al., 2004; Anderson and Hope, 2009) and they confirm the idea that neuroticism and AS must be considered as direct cognitive risk-factors of anxiety disorders.

Our analyses of this model also showed that other personality dimensions appeared to act with neuroticism to have a significant role in the predictive model of anxiety symptoms. Notably, high neuroticism and low levels of extraversion predict GAD symptoms, supporting studies demonstrating that GAD is characterized by high-levels of self-focus attention (Mor and Winquist, 2002). Self-focus attention reflects to an awareness of self-reference or self-generated information (Ingram, 1990), which is conceptually opposed to extraversion. Furthermore, high levels of neuroticism and conscientiousness predicted SP symptoms. These data are in accordance with the previous suggestions of Bienvenu et al. (2004) according to which children with high conscientiousness would show high levels of self-discipline and achievement striving. Those characteristics would predispose children to develop high representations of social situations and to avoid those situations consequently.

Surprisingly, analyses revealed that conscientiousness considered alone did not predict the occurrence of SP symptoms. The same result was observed for extraversion in the model predicting GAD, revealing the importance of the connection of these personality traits with neuroticism. Finally, high levels of neuroticism and AS are coupled with low levels of agreeableness in the predicting model of depressive symptoms, raising the importance of emotion regulation difficulties in depression.

The last model tested in our study aimed to evaluate the impact of personality traits on AS dimensions and the impact of these dimensions on the investigated symptoms. We first observed that associations between personality traits and anxiety symptoms confirmed the observations made in the second model presented in this study. Second, we found that low levels of agreeableness significantly predicted the presence of physical, cognitive and control concerns about anxiety symptoms, confirming the major role of agreeableness in the development of concerns about anxiety symptoms. Nevertheless, we found that the social dimension of the AS was not predicted by any of the personality dimensions, suggesting it may depend on other vulnerability factors. Surprisingly, other personality dimensions appeared to have a significant role in the predicting model of those dimensions. Notably, fear of losing control concerns were associated to low levels of openness and high levels of conscientiousness. Interestingly, while correlated positively, the four distinct dimensions of AS did not separately influence anxiety symptoms, highlighting the importance to consider AS as a unitary concept in the development of a predicting and hierarchical model of anxiety symptoms. However, these results may be directly connected to the factor structure of the CASI used in our study. Indeed, we used a four-factors structure (social, physical, cognitive, and control) as suggested by the research of Stassart and Etienne (2014) conducted on French-speaking children. However, the factor structure of AS scales set off numerous debates (Stassart and Etienne, 2014). Indeed, while the majority of authors agreed to distinguish the fear of physiological symptoms from mental, social and control concerns, their subdivision into three dimensions is subtler, with social and control concerns sometimes considered as a unified dimension. Further studies should therefore be conducted to investigate the precise factorial structure of the CAS.

It is also important to consider the results obtained in this study, since participants came from a community sample of children reporting no neurological or developmental disorders and reporting non-clinical levels of anxiety. This study should be replicated in children suffering from clinical anxiety disorders to confirm the role of personality traits and AS in specific fears and anxieties in children. Indeed, the replication of the hierarchical model developed by Sexton et al. (2003) in clinical samples allowed us to draw some consequent conclusions about the model.

A limitation of this study is that we decided to use self-reported inventories for anxiety similar to that used for personality. We may question the validity of self-reported variables in children because of their limited cognitive abilities and their possible lack of engagement toward those methods. However, Measelle et al. (2005) found that children as early as 5 years of age, are able to describe themselves reliably on a self-reported Big Five scale and that their ratings were increasingly consistent with evaluations by parents and teachers. These data confirm that young children already have a coherent, stable and valid perception of themselves and confirming previous studies enhancing the validity to investigate personality dimensions in this population which has also long been debated (Tackett et al., 2008, 2012; Soto and John, 2014). Future studies should also multiply the number of informants to assess children’s anxiety and depression levels.

In conclusion, this study aimed to draw a hierarchical model of the associations between personality traits, AS and anxiety symptoms. In Sexton et al. (2003) model developed on adults, AS was considered as a specific vulnerability factor that depends on a subordinate vulnerability factor, neuroticism, and both are thought to promote the presence of anxiety symptoms. Since our second and extended hierarchical model seems to be the one that best suits the data collected in our sample of children aged between 8 and 12, we can conclude that both high levels of neuroticism and AS acts as vulnerability factors in the onset of the vast majority of anxiety symptoms encountered in pediatric population. However, contrary to our expectations, we failed to demonstrate a hierarchical relationship between these two constructs. Our analyses suggest that, in children, AS would depend more on low levels of agreeableness which underlines the importance of PA. Our study also allows to extend the model of Sexton et al. (2003) by showing that neuroticism and AS act in collaboration with conscientiousness and extraversion in the manifestations of anxiety symptoms. Finally, our data suggests the importance of considering AS as a unitary construct in the predicting model of anxiety symptoms.

Altogether, our results suggest that AS and other trait-like factors may act as risk factors in the development of later anxiety disorder. However, to confirm this and to support previous data demonstrating this (Weems et al., 2007; Schmidt et al., 2010), it seems crucial to conduct a longitudinal study focusing on these aspects. If such results can be obtained in future work, researchers’ attention should be drawn on the construct of AS in association with certain personality dimensions; notably in the development of interventions that would help children detect and recognize their symptoms of anxiety and help them to interpret them correctly.

## Author Contributions

EW and MR had the initial ideas. EW collected and wrote the drafts and the final manuscript. EW and KH analyzed the data. MR reviewed the several drafts of the manuscript. KEB, WB, and LL revised the manuscript. All authors approved the final version of the manuscript.

## Conflict of Interest Statement

The authors declare that the research was conducted in the absence of any commercial or financial relationships that could be construed as a potential conflict of interest.
