# Supplementary material for: Examining the Hierarchical Influences of the Big-Five Dimensions and Anxiety Sensitivity on Anxiety Symptoms in Children
Source: Front Psychol. 2019 Jun 4;10:1185. doi: 10.3389/fpsyg.2019.01185 (PMC6558314; doi:10.3389/fpsyg.2019.01185)
Supplement: Supplementary file 1 [file Data_Sheet_1.PDF]

**Table 1:** Pearson correlation matrix and univariate summary statistics.

| | 1 | 2 | 3 | 4 | 5 | 6 | 7 | 8 | 9 | 10 | 11 | 12 | 13 | 14 | 15 | 16 | 17 | 18 |
|---|---|---|---|---|---|---|---|---|---|---|---|---|---|---|---|---|---|---|
| **1. CASI** | | | | | | | | | | | | | | | | | | |
| **2. Physical** | .910** | | | | | | | | | | | | | | | | | |
| **3. Social** | .735** | .650** | | | | | | | | | | | | | | | | |
| **4. Cognitive** | .559** | .354** | .354** | | | | | | | | | | | | | | | |
| **5. Control** | .780** | .716** | .355** | .521** | | | | | | | | | | | | | | |
| **6. STAIC** | .449** | .396** | .346** | .422** | .414** | | | | | | | | | | | | | |
| **7. O** | -.065 | -.114 | -.019 | -.042 | -.123 | -.039 | | | | | | | | | | | | |
| **8. C** | -.028 | -.054 | -.008 | -.067 | .010 | -.052 | .501** | | | | | | | | | | | |
| **9. E** | .020 | .008 | -.014 | -.032 | -.017 | -.063 | .509** | .306** | | | | | | | | | | |
| **10. A** | -.207** | -.207** | -.038 | .266** | -.186** | -.211** | .410** | .454** | .377** | | | | | | | | | |
| **11. N** | .135 | .079 | .191** | .169* | .054 | .517** | .014 | -.112 | .041 | -.196** | | | | | | | | |
| **12. RCADS** | .465** | .401** | .298** | .451** | .415** | .770** | .040 | .035 | -.087 | .035 | .352** | | | | | | | |
| **13. SP** | .429** | .371** | .306** | .392** | .438** | .777** | -.027 | .074 | -.047 | .074 | .390** | .787** | | | | | | |
| **14. PD** | .489** | .469** | .286** | .487** | .363** | .646** | .007 | -.014 | -.044 | -.014 | .290** | .766** | .607** | | | | | |
| **15. GAD** | .363** | .286** | .272** | .387** | .375** | .684** | -.082 | -.049 | -.156* | -.049 | .293** | .821** | .647** | .626** | | | | |
| **16. OCD** | .361** | .340** | .244** | .341** | .379** | .487** | .087 | .183** | -.018 | .183** | .202** | .717** | .556** | .589** | .766** | | | |
| **17. SAD** | .417** | .381** | .302** | .393** | .353** | .625** | -.036 | .016 | -.102 | .016 | .334** | .720** | .546** | .766** | .676** | .473** | | |
| **18. Depression** | .445** | .376** | .298** | .424** | .368** | .724** | .096 | .042 | .032 | -.195** | .382** | .821** | .742** | .658** | .601** | .575** | .597** | |
| **Min** | 17 | 7 | 3 | 4 | 3 | 22 | 29 | 29 | 33 | 28 | 17 | 2 | 2 | 0 | 0 | 0 | 0 | 0 |
| **Max** | 37.95 | 20 | 9 | 8 | 12 | 47 | 60 | 53 | 59 | 55 | 47 | 77 | 26 | 16 | 15 | 13 | 12 | 17 |
| **Mean** | 25.91 | 10.33 | 5.73 | 5.2 | 6.48 | 33.8 | 46.69 | 42.14 | 46.53 | 42.47 | 32.9 | 30.6 | 9.93 | 4.69 | 6.09 | 4.67 | 4.87 | 6.27 |
| **SD** | 5.74 | 3.12 | 1.59 | 1.31 | 1.99 | 7.12 | 7.62 | 6.5 | 6.78 | 7.43 | 7.81 | 18.19 | 5.43 | 4.67 | 4.18 | 3.72 | 3.51 | 3.88 |

*O= Openness; C= Conscientiousness; E= Extraversion; A= Agreeableness; N= Neuroticism) (SP= Social Phobia; PD= Panic Disorder; GAD= Generalized Anxiety Disorder; OCD = Obsessive-Compulsive Disorder; SAD = Separation Anxiety Disorder*; ** for p-values < .05; ** for p-values < .01*

**Table 2:** First hypothesized model investigating the hierarchical influences of Neuroticism and Anxiety Sensitivity (AS) on Panic Disorder (PD), Generalized Anxiety Disorder (GAD) and Obsessive-Compulsive Disorder (OCD) symptoms (standardized path coefficients, R-squared and Adjusted R-squared).

| **Factor** | **Predictor** | **β** | **$R^2$** | **Adjusted $R^2$** |
|---|---|---|---|---|
| Anxiety Sensitivity | Neuroticism | .148 | .022 | .016 |
| PD | Anxiety Sensitivity | .441** | .298 | .288 |
| | Neuroticism | .260** | | |
| GAD | Anxiety Sensitivity | .343** | .233 | .223 |
| | Neuroticism | .291** | | |
| OCD | Anxiety Sensitivity | .357** | .182 | .172 |
| | Neuroticism | .187* | | |

** for p-values <.05; ** for p-values < .01*

**Table 3:** Second hypothesized model investigating the hierarchical influences of the Big Five dimensions and Anxiety Sensitivity on anxiety symptoms (standardized path coefficients, R-squared and Adjusted R-squared)

| Factor | Predictor | β | $R^2$ | Adjusted $R^2$ |
|---|---|---|---|---|
| Anxiety Sensitivity | Openness | -.214 | .125 | .089 |
| | Conscientiousness | .178 | | |
| | Extraversion | .153 | | |
| | Agreeableness | -.305* | | |
| | Neuroticism | .106 | | |
| SP | Openness | -.060 | .364 | .333 |
| | Conscientiousness | .207* | | |
| | Extraversion | -.130 | | |
| | Agreeableness | -.026 | | |
| | Neuroticism | .472** | | |
| | Anxiety Sensitivity | .310** | | |
| PD | Openness | .057 | .239 | .201 |
| | Conscientiousness | .047 | | |
| | Extraversion | -.061 | | |
| | Agreeableness | -.071 | | |
| | Neuroticism | .336** | | |
| | Anxiety Sensitivity | .285** | | |
| GAD | Openness | .128 | .277 | .242 |
| | Conscientiousness | -.012 | | |
| | Extraversion | -.235* | | |
| | Agreeableness | -.036 | | |
| | Neuroticism | .355** | | |
| | Anxiety Sensitivity | .305** | | |
| OCD | Openness | .024 | .221 | .183 |
| | Conscientiousness | .350** | | |
| | Extraversion | -.063 | | |
| | Agreeableness | -.166 | | |
| | Neuroticism | .198* | | |
| | Anxiety Sensitivity | .252* | | |
| SAD | Openness | .117 | .309 | .275 |
| | Conscientiousness | .070 | | |
| | Extraversion | -.163 | | |
| | Agreeableness | -.109 | | |
| | Neuroticism | .411** | | |
| | Anxiety Sensitivity | .271** | | |
| DEPRESSION | Openness | .191 | .336 | .303 |
| | Conscientiousness | .095 | | |
| | Extraversion | -.086 | | |
| | Agreeableness | -.067* | | |
| | Neuroticism | .408** | | |
| | Anxiety Sensitivity | .328** | | |

*SP= Social Phobia; PD= Panic Disorder; GAD= Generalized Anxiety Disorder; OCD= Obsessive-Compulsive Disorder; SAD = Separation Anxiety Disorder; * for p-values <.05; ** for p-values < .01*

**Table 4:** Third hypothesized model investigating the hierarchical influence of the Big Five dimensions and of AS dimensions (standardized path coefficients, R-squared and Adjusted R-squared).

| Factor | Predictor | β | $R^2$ | Adjusted $R^2$ |
|---|---|---|---|---|
| Cognitive | Openness | -.058 | .147 | .112 |
| | Conscientiousness | .142 | | |
| | Extraversion | .132 | | |
| | Agreeableness | -.382** | | |
| | Neuroticism | .137 | | |
| Physical | Openness | -.217 | .124 | .088 |
| | Conscientiousness | .139 | | |
| | Extraversion | .194 | | |
| | Agreeableness | -.316* | | |
| | Neuroticism | .068 | | |
| Control | Openness | -.322** | .127 | .092 |
| | Conscientiousness | .245* | | |
| | Extraversion | .166 | | |
| | Agreeableness | -.284** | | |
| | Neuroticism | .012 | | |
| Social | Openness | -.110 | .050 | .011 |
| | Conscientiousness | .088 | | |
| | Extraversion | -.004 | | |
| | Agreeableness | -.054 | | |
| | Neuroticism | .189 | | |
| | Openness | -.054 | | |
| SP | Conscientiousness | .205* | .368 | .320 |
| | Extraversion | -.129 | | |
| | Agreeableness | .025 | | |
| | Neuroticism | .462** | | |
| | Cognitive | .054 | | |
| | Physical | .135 | | |
| | Control | .078 | | |
| | Social | .131 | | |
| PD | Openness | 041 | .254 | .198 |
| | Conscientiousness | .046 | | |
| | Extraversion | -.064 | | |
| | Agreeableness | -.042 | | |
| | Neuroticism | .315** | | |
| | Cognitive | .199 | | |
| | Physical | .108 | | |
| | Control | .003 | | |
| | Social | .086 | | |
| GAD | Openness | .141 | .305 | .253 |
| | Conscientiousness | -.037 | | |
| | Extraversion | -.235* | | |
| | Agreeableness | -.013 | | |
| | Neuroticism | .345** | | |
| | Cognitive | .185 | | |
| | Physical | -.073 | | |
| | Control | .221 | | |
| | Social | .102 | | |

| | | | | |
|---|---|---|---|---|
| OCD | Openness | .054 | .241 | .184 |
| | Conscientiousness | .330* | | |
| | Extraversion | -.083 | | |
| | Agreeableness | -.139 | | |
| | Neuroticism | .212* | | |
| | Cognitive | .048 | | |
| | Physical | .111 | | |
| | Control | .182 | | |
| | Social | -.015 | | |
| SAD | Openness | .133 | .345 | .296 |
| | Conscientiousness | .058 | | |
| | Extraversion | -.150 | | |
| | Agreeableness | -.124 | | |
| | Neuroticism | .390** | | |
| | Cognitive | .034 | | |
| | Physical | .012 | | |
| | Control | .136 | | |
| | Social | .229* | | |
| DEPRESSION | Openness | .186 | .325 | .274 |
| | Conscientiousness | .098 | | |
| | Extraversion | -.084 | | |
| | Agreeableness | -.065 | | |
| | Neuroticism | .400** | | |
| | Cognitive | .090 | | |
| | Physical | .150 | | |
| | Control | .103 | | |
| | Social | .047 | | |

*SP= Social Phobia; PD= Panic Disorder; GAD= Generalized Anxiety Disorder; OCD= Obsessive-Compulsive Disorder; SAD = Separation Anxiety Disorder; * for p-values <.05; ** for p-values < .01*
